# About differences in the availability of child and adolescent psychiatric hospital beds across Europe and possible implications for mental health care. Are more or less hospital beds the solution to the youth mental health crisis? Based on a debate at the 21st International Congress of the European Society for Child and Adolescent Psychiatry, Strasbourg, June 30, 2025

**DOI:** 10.1007/s00787-025-02902-7

**Published:** 2025-10-27

**Authors:** Joerg M. Fegert, Isabel Boege, Dario Calderoni, Diane Purper-Ouakil, Emily Sitarski, Benedetto Vitiello

**Affiliations:** 1 Department of Child and Adolescent Psychiatry, Psychosomatics and Psychotherapy, University Hospital Ulm, Steinhövelstraße 5, Ulm, 89075 Germany; 2 German Center for Mental Health (DZPG), partner site Ulm, Steinhövelstraße 5, Ulm, 89075 Germany; 3 Competence Center Public Child Mental Health, partner site Ulm, Steinhövelstraße 5, Ulm, 89075 Germany; 4 Department of Child and Adolescent Psychiatry, Psychosomatics and Psychotherapy, ZfP Südwürttemberg, Weingartshofer Strasse 2, Ravensburg, 88214 Germany; 5 Department of Child and Adolescent Psychiatry, Psychosomatics and Psychotherapy, Medical University of Graz, Auenbruggerplatz 34, 8036 Graz, Austria; 6 Department of Human Neuroscience, Section of Child and Adolescent Neuropsychiatry, Policlinico Umberto I- Sapienza University of Rome, Rome, Italy; 7 Child and Adolescent Psychiatry, University Hospital of Montpellier, Saint Eloi Hospital, Montpellier, France; 8 CESP, INSERM U1178, Centre de recherche en Épidémiologie et Santé des Populations, Villejuif, France; 9 Section of Child and Adolescent Neuropsychiatry, University of Turin, Turin, Italy; 10 Dept. of Mental Health, Johns Hopkins University Bloomberg School of Public Health, Baltimore, MD USA

**Keywords:** Youth mental health crisis, Child and adolescent psychiatric hospital beds, Europe, Mental health, Hospitalization, Child and adolescent mental health care, Behavioral outcomes, Psychopathology, Innovative youth mental health services

## Abstract

A number of indicators point to a decline in youth mental health globally over the past 15 years. Access to medical treatment and assistance is becoming increasingly difficult as the social divide is widening due to an imbalance between supply and demand. Hospitalization remains a critical yet costly component of child and adolescent mental health care, reserved for the most severe cases. While necessary in some situations, inpatient treatment carries substantial drawbacks, including family separation and social disruption. This paper discusses if increasing hospital beds across Europe can be the solution to the youth mental health crises by examining differences in the availability of child and adolescent psychiatric hospital beds across Europe. International comparisons reveal striking heterogeneity in hospital beds availability, which does not correlate with improved mental health and behavioral outcomes in young people at the population level. Furthermore, the data does not suggest that this heterogeneity reflects the need to address different levels of psychopathology. Besides the limitations of the currently available data, evidence suggest that effective outpatient and home-based interventions can reduce hospital dependence without compromising treatment efficacy. The COVID-19 pandemic highlighted both the vulnerability of traditional services and the growing role of digital mental health support, which adolescents increasingly access via online platforms. Emerging stepped-care and community-based models, including lay-provider interventions, demonstrate potential to expand reach while conserving specialized resources. Given scarce capacity, European-wide recommendations are needed to ensure equitable access to acute care, while integrating innovative, digitally supported, low-threshold services into youth mental health systems.

## Introduction

A number of indicators point to a decline in youth mental health globally over the past 15 years [1]. The COVID-19 pandemic further worsened the situation by interrupting social and educational activities, elements of great relevance for a healthy psychosocial development. Parallel disruption of mental health services took place, even worsening the psychosocial effect [2]. In this global youth mental health crisis, access to medical treatment and assistance is in addition becoming increasingly difficult, especially for the low-income families as well as for families without connections, resources, or influence. The social divide is widening due to an imbalance between supply and demand, and the impact of cumulative inequality in risk and protective factors worsens [3]. The effects of the *inverse care law* are becoming apparent, with those most in need of help finding it increasingly difficult to access care [4].

In the context of the ongoing youth mental health crisis, it can be worth examining the resources available to the youths with severe psychiatric disturbances requiring acute hospital care. In particular, it can be informative to consider the availability of hospital beds across European countries and discuss possible implications for improving mental health care. In practical terms: can increasing hospital beds be part of a solution to the youth mental health crisis?

## Psychiatry hospital beds for youth across Europe

Although limited in number and methods, the available data show a wide variability in the estimated number of psychiatric hospital beds per 100,000 youth (defined as of legally minor age, usually under 18 years) across 28 countries in Europe, ranging from 64.0 in Germany to 1.1 in Sweden [5]. There is also substantial variability within the same country. Thus, within Germany, for 2019, the estimated rate was 34.9 per 100,000 youth in Bavaria and 106.4 in Saxony-Anhalt in 2019 [6]. For 2022, rates of 32 in Bavaria and 105 in Thuringia were reported [7, 8].

Table 1 contrasts the top four countries with the four bottom ones (among countries with population of 5 million or more). The heterogeneity between countries is not specific to child and adolescent psychiatry beds, but can be also represented in the availability of general psychiatry beds and that of any hospital beds (Table 1). There appears to be some correspondence between youth and adult bed availability, with Germany, Netherlands, and Czechia having high rates of both youth and general psychiatric beds as compared with the European average. On the other side, Italy and Spain have very low rates of both youth and general psychiatric beds.

**Table 1:** Countries with the highest and lowest estimated number of youth psychiatric hospital bed

	Highest rates of hospital beds				Lowest rates of hospital beds			
	Germany	Netherlands	Czechia	Finland	Sweden	Spain	Italy	Greece
Youth psychiatry hospital beds^a^ N/100,000	64.0	56.6	34.6	32.3	1.2	2.4	3.2	3.2
General psychiatry hospital beds^b^ N/100,000	128	85	92	57	42	39	9	72
All hospital beds N/100,000^c^	766.4	231.1	640.7	259.5	187.4	288.1	304.1	424.1
Mental Health expenditure as % of total health government expenditure^d^	11	10.7	2.9	3.9	10	5	5	4.4

Notes:

^a^Data extracted from Signorini et al. 2017 [5] (collected in 2014–2015). Countries of at least 5 million population with number psychiatric hospital beds per 100,000 minors, respectively greater than 30 or lower than 5

^b^From Hospital beds from Eurostat (2018 data): https://ec.europa.eu/eurostat/web/products-eurostat-news/-/edn-20201009-1 (accessed August 5, 2025) (https://ec.europa.eu/eurostat/en/web/products-eurostat-news/-/edn-20191009-1) (accessed August 7, 2025). Closely comparable figures (N/1,000) can be found in the OECD Health Statistics 2023 for the year 2021: Germany: 7.8, Netherlands: 3.0, Czechia: 6.7, Finland: 2.8, Sweden: 3.0, Spain 3.0, Italy: 3.1, and Greece: 3.3

^c^From: Eurostat: Healthcare resource statistics - beds (data 2023) (https://ec.europa.eu/eurostat/statistics-explained/index.php?title=Healthcare_resource_statistics_-_beds&oldid=653301) (accessed 25/07/25)

^d^From the WHO Global Health Observatory: Mental Health expenditure by country as percentage of total government health expenditures (data 2011) https://www.who.int/data/gho/data/indicators/indicator-details/GHO/government-expenditures-on-mental-health-as-a-percentage-of-total-government-expenditures-on-health-(-) (accessed 25/07/2025)

It should be noted that, across the EU, the general trend has been towards a gradual decrease in psychiatric beds per capita over the past decades, the rate being 73 beds per 100,000 inhabitants in 2018 vs. 79 beds in 2004 [9]. By contrast to this development, a significant increase in psychiatric hospitalizations for female adolescents with anxiety, eating disorders and depression has been observed in the past few years post Covid-19 [10].

At the same time, the proportion of the public health expenditure devoted to mental health does not seem to be associated with the availability of psychiatric beds, as suggested by the fact that both the countries with the highest (Germany) and lowest (Sweden) number of beds per population spend about 10% of their health budget on mental health (Table 1). This indicates that some countries, like Sweden, allocate more resources to outpatient rather than inpatient services compared with other countries like Germany, the Netherlands, Czechia, and Finland.

## Hospital beds availability and global indexes of mental health in youth

Are there any apparent correspondences between hospital beds availability and global indexes of mental health? As important as this question seems, the lack of validated measures, that are consistently and prospectively applied across countries, prevents assessing the possible presence of links between mental health indexes and availability of psychiatric hospital beds. A few reports on international psychopathology, however, can provide tentative views and be hypothesis-stimulating.

Is there any evidence that countries that devote more resources to inpatient services for youth do so because there is more psychopathology in their population than in those of countries that allocate fewer resources? This hypothesis cannot be discarded a priori when considering that there is heterogeneity in the prevalence of mental disorders in the world. A systematic review and meta-analysis, including data from 27 countries world-wide, reported a prevalence of mental health disorders of 13.4% (CI 95% 11.3–15.9), with substantial and statistically significant heterogeneity [11].

Data specifically focused on Europe are rather limited. A study using the Strength and Difficulties Questionnaire (SDQ) completed by parents and/or teachers reported that overall 12.8% of children aged 6–11 years had a probable mental health disorder, with between-country variability [12]. Germany (12.8%) and the Netherlands (11.2%) were among the countries with highest rates while the lowest rate was in Italy (7.8%). In this study, the country variability was accounted for by differences in sociodemographics and parental psychological distress. Other data, however, do not suggest that countries with high hospital bed rates have greater indexes of general psychopathology. A screening of 12–18 years olds using self-report SDQ found percentages of deviant total difficulties scores of (among others) 10.0% in Germany, 10.4% in the Netherlands, 20.7% in Czechia, 15.1% in Spain, 21.0% in Greece, and 12.8% in Sweden [13]. Estimates of psychopathology among children and adolescents, using the CBCL total problem score in 44 societies worldwide, reported an overall mean score of 24.0% (SD 6.74) [14], with rates of 16.0% in Germany, 20.0% in the Netherlands, 18.0% in Finland, 23.0% in Italy, 28.0% in Greece, and 13.0% in Sweden (visually estimated from Fig. 1 of [14]).

It may well be that different countries have a different focus on treatments being offered. While some countries focus more on ambulatory care, others focus more on inpatient care. In 2023–2024, for example, 3,130 patients were admitted to a psychiatric inpatient unit in England for more than 1 week [15] serving a population of 14,403,544 under 18 years [16], while in Germany there were 61,942 hospital admissions [17] for a population of 13,969,984 under 18 years. However, there seems to be a beginning of change in treatment options in Germany, as a greater focus on intensive outpatient treatment has been placed since 2018, when inpatient equivalent home treatment has been incorporated by stakeholders into the possible treatment options. Especially in child and adolescent psychiatry this approach is well justified, as it enables greater parental involvement. There is evidence that treatment outcomes in home treatment settings are equally effective, and, even more important, often more sustainable than inpatient care [18].

A parallel question is whether a greater investment in psychiatric inpatient services is accompanied by lower negative outcomes at the population level. Again, despite the importance of the question, the absence of systematically collected and validated psychiatric outcome measures prevents addressing this issue properly. One could explore general rates of self-reported good health, or indexes of functioning and mortality, such as suicide rate, mortality from road accident (as possible index of reckless, impulsive behavior), or avoidable mortality. In addition, the prevalence of “youth Not in Education, Employment, or Training (NEET)” can be considered an overall index of functioning among youth. A visual inspection of these rates by countries with highest vs. the lowest youth psychiatric beds does not suggest that these indicators are more favorable in the countries with the highest availability in psychiatric hospital beds for youth (Table 2). Thus, the youth suicide rates in the four countries with the lowest number of beds is not lower than those seen in the four countries with the highest number of beds. Sweden, with the lowest number of hospital beds (1.2 per 100,000), has had a youth suicide rate of 3.99 per 100,000 among 10 to 19 years old/year, while Finland having one of highest number of psychiatric beds for youth (32.3 per 100,000), reported 4.99 per 100,000. Moreover, the proportion of young people (age 16–29 years) who reported to perceive themselves to be in “good or very good health” in 2024 was 97.7% in Greece, 95.3% in Italy and 91.7% in Spain, countries with the smallest number of hospital beds. These rates are comparable to those reported in Germany (87.8%), Netherlands (80.8%), and Czechia (93.6%), which have the highest number of beds. NEET may be less prevalent in the countries with highest number of beds, but this parameter is likely influenced also by cultural and socio-economic variables beyond mental health issues [19].

**Table 2:** Possible indexes of global mental health in countries with the highest and lowest estimated number of youth psychiatric hospital bed. Notes:

	Highest rates of hospital beds				Lowest rates of hospital beds			
	Germany	Netherlands	Czechia	Finland	Sweden	Spain	Italy	Greece
Youth psychiatry hospital beds^a^ N/100,000	64.0	56.6	34.6	32.3	1.2	2.4	3.2	3.2
Suicide age 10–19 yers N/100,00/year^b^	2.75	2.39	3.90	4.96	3.99	1.47	1.50	1.30^d^
Life expectancy^c^ (years)	80.2	81.4	77.2	81.9	83.1	83.1	82.7	80.2
Road injuries mortality for age 15–17 N/1 million/year^e^	34	15	33	45	19	17	38	50
Share (%) of youth aged 16–29 years self-perceived to be in good or very good health^f^	87.7	80.8	93.6	79.3	76.3	91.7	95.3	97.7
NEET^g^, %	8.5	4.9	8.6	10.0	6.3	12.0	15.2	14.2

^a^From Signorini 2017 [5] (data collected in 2014–2015)

^b^Glenn CR, Kleiman EM, Kellerman J, Pollak O, Cha CB, Esposito EC, Porter AC, Wyman PA, Boatman AE (2020) Annual Research Review: A meta-analytic review of worldwide suicide rates in adolescents. J Child Psychol Psychiatry 61(3):294–308. doi: 10.1111/jcpp.13106. ^c^From OECD Health at a Glance, 2023, age-standardized for avoidable mortality, OECD (2023), Health at a Glance 2023: OECD Indicators, OECD Publishing, Paris, 10.1787/7a7afb35-en

^d^Age 10–24 yrs (calculated over the years 2000–2009), from: Bacopoulou F, Petridou E, Korpa TN, Deligeoroglou E, Chrousos GP (2015) External-Cause Mortality among Adolescents and Young Adults in Greece over the Millennium’s First Decade 2000–09. J. Public Health 37:70–77

^e^European Road Safety Observatory. Traffic Safety Basic Facts – 2018 – Youngsters (15–17). Available at: https://road-safety.transport.ec.europa.eu/document/download/25694782-92b4-4b96-be30-c6e8e8f28548_en?filename=bfs2018_youngsters.pdf (accessed August 6, 2025)

^f^From EUROSTAT, data from 2024, https://ec.europa.eu/eurostat/statistics-explained/index.php?title=Young_people_-_health

^g^NEET (Not in Education, Employment or Training), age 15–19 years, 2024, Eurostat

https://ec.europa.eu/eurostat/statistics-explained/index.php?title=Statistics_on_young_people_neither_in_employment_nor_in_education_or_training

## Conclusion and discussion

Hospital care is one of the components of mental health services, though arguably the most expensive one. While it provides care to just a small fraction of all youth with psychiatric needs in most countries, it treats the most severe patients, who cannot be managed in less restrictive settings. Besides the higher costs, there are other drawbacks and intangible costs to be considered. Hospitalization interrupts the usual educational and social activities of the youth; it removes them from friends and family; and reintegration into the social and educational settings after hospital discharge can be difficult [20]. In the context of separation from family due to psychiatric hospitalization, reference should also be made to the United Nations Convention on the Rights of the Child [21]. Article 9 stipulates that a child may only be separated from their parents under certain conditions and if it is in the child’s best interest. Examples include cases of child abuse and neglect. Children separated from their families must enjoy special protection. Therefore, psychiatric inpatient measures and hospitalizations involving separation from family require special consideration with regard to the UN Convention on the Rights of the Child. The negative consequences of family separation for children and the necessity of psychiatric hospitalization must be carefully weighed. Moreover, hospitalization can have uninteded, negative effects on patients by exposing them to peers with other manifestations of psychopathology with the risk of “contagion” by acquiring new symptoms and dysfunctional behaviors [22, 23].

Therefore, determining the optimal utilization of psychiatric inpatient resources is of high public health relevance. Alternative treatments in an outpatient setting, even in patients with severe impairment, should always be considered. Furthermore, a meta-analysis demonstrated that home-based treatment is not less effective than inpatient hospitalization, suggesting that once acute stabilization is achieved, many adolescents might benefit from ongoing care in a safe home environment rather than prolonged inpatient stays [24]. Hospital use is interdependent with outpatient and day-hospital services. The need for hospital beds and duration of inpatient stay are tightly dependent on the type, quality and efficiency of the outpatient and day hospital services. When outpatient services are efficient and easily accessible, fewer youth are likely to require hospitalization, while the opposite will happen in case of deficiencies in outpatient services. During and after the COVID-19 pandemic, the outpatient services were often disrupted, thus putting more pressure on hospitals. Home treatment, as an alternative to inpatient treatment, was offered even during the COVID-19 pandemic and in those places where it was available, proved to be one of the most widely used and in-demand therapeutic options during the COVID-19 pandemic, while inpatient resources were underutilised. Home treatment included a 60 to 90-minute home visit each day (including weekends) for 6 weeks, offered by a multi-professional team. Before commencing treatment, an individual treatment plan was developed co-jointly with the family and patient. Elements offered within the 6 weeks of intensive home treatment were individual therapy, family therapy, parental coaching, occupational therapy, social work, activation, physiotherapy and hippotherapy [25].

In other regions without home treatment services in place, the structural overload of the healthcare system during the pandemic, combined with increased mental stress among young people, has led young people to primarily seek low-threshold mental health support in the digital space, e.g., through online content, by online communities or through free, accessible online counseling. They turn to online services or their peers for help, rather than traditional healthcare providers like general practitioners, specialists, or emergency rooms. Compared to other generations, the younger generation’s trust in the medical profession has declined significantly across Europe [26]. Unlike traditional healthcare services, online mental health services reach the target group of adolescents and young adults thanks to their low threshold and uncomplicated access. In 2023, the YouTube platform recorded 35 billion views of mental health videos worldwide [27]. Moreover, in the same year, YouTube noted a 25% year-on-year increase in the number of videos uploaded related to mental health [27]. This is a major generational change that, while raising concerns about misinformation and “psychological contagion”, offers also opportunity for psychoeducation and self-help [28, 29].

More of the same cannot be the solution to the current problems. We need to think more carefully about how we develop interfaces between centers providing high-quality care in hospitals and medical practices and the demand of young people on the internet. A stepped care approach can maximize the effectiveness of outpatient and day hospital services, leaving hospitalization as the last option to use, especially to address acute risk to health of the youth or others. However, it is also time to consider radically new approaches to mental health care. For example, a psychotherapeutic model from Kenya is establishing care services that rely on trained, peer lay providers to deliver brief, de-stigmatized, evidence-based interventions. Experts (psychologists and psychiatrists), who are rare and expensive, are only called upon in complex cases that require more intensive care, following a three-step, tiered model [30]. Initial analyses have shown that this approach does not result in worse outcomes than traditional individual therapy [31]. At the same time, however, it reaches many more young people and enables them to participate in normal life.

In this respect, given the scarce resources available for outpatient and inpatient psychiatric/psychotherapeutic treatment, it is important to consider how we can put all these elements together, how we can implement and integrate intelligent, digitally supported stepped care approaches with low-threshold chat counseling services, screening, easily understandable psychoeducation, health information, and everyday tips up to specialized care. These digital services could be used at all levels of prevention (universal, selective, and indicated) by offering young people low-threshold answers, access to healthcare and information before they even come into contact with psychiatric/psychotherapeutic services. The services could also provide individual counseling to at-risk young people and support them in transitioning from indicated prevention to early intervention on their way to specialized care. Concluding, online mental health services such as ‘Krisenchat’ in Germany — a chat service for young people staffed by qualified counselors — or ‘The 3114’ - a French national number offering 24/7 access to professionals trained in assessing and counseling for suicidal ideation and behaviour for all age groups and for close ones and professionals [32] - could play a central role as intelligent gatekeepers in a stepped care approach.

The wide variability across Europe in the number of psychiatric hospital beds for youth likely reflects differences in the overall organization and efficiency of the mental health services [5]. Culture and tradition of psychiatric care varies across societies, and these factors influence the way psychiatrist and other mental health providers are trained and services are organized and funded.

The extreme variability in hospital beds for youth, ranging from an estimated 1.2 beds per 100,000 youth in Sweden to 64.0 in Germany is not accompanied by detectable differences in mental health and behavioral outcomes at the population level (Table 1). Based on the available data, there are no indications that these differences in psychiatric hospital resources results in better mental health outcomes, nor are there data suggesting that the heterogeneity reflects the need to address different levels of psychopathology (Table 2). However, it must be recognized that there are limitations in the currently available data and a lack of validated and systematically collected measures across countries. “Hard” outcomes such as suicide or NEET status are likely influenced by multiple factors, such as economic stability, education, healthcare, housing, social relationships, and physical environment, in addition to mental health [33]. In this case, therefore, lack of evidence cannot be taken as evidence of absence.

It is noteworthy that a lower number of psychiatry hospital beds is not necessarily indicative of a lower investment in mental health. Thus, the share of health funds devoted to mental health is similar (about 10%) in Sweden, the country with the lowest number of beds, as in Germany, the country with the highest number of beds (Table 1). This may help explain the lack of detectable differences in outcomes by suggesting that more patients could be managed without hospitalization.

One issue about the differences in the number of psychiatric beds for youth (see Table 2) is whether and to which extent youth in need of psychiatric hospitalization are hospitalized in non-psychiatric beds, such as pediatrics or general medicine wards, or in adult psychiatry beds. In Italy, a country with one of the lowest rate of youth psychiatric beds, it has been reported that as many as six out of 10 adolescents aged 15–18 years needing hospitalization may be hospitalized in adult psychiatry or non-psychiatric beds [34]. The “overflow” of youth from adolescent into adult psychiatry or pediatric beds can be taken as an index of insufficient number of dedicated hospital beds.

Another consideration is about psychiatric hospitalizations due to family and social contexts that are grossly inadequate and do not provide the conditions for outpatient treatment. The extent of these “social hospitalizations” has not been systematically tracked, but it may be substantial in some areas and may contribute to longer hospitalizations and bed shortages.

The optimal duration of the hospitalization is also a highly relevant aspect of inpatient care. Duration will obviously depend on the type of psychopathology and the individual characteristics of the patients and their social context [35]. But, also on the presence and provision of intensity and method differentiated ambulatory care settings. For the home treatment setting it has been proven that an inpatient hospital stay can be shorted up to 22–23 days when intensive outpatient care can be provided [25]. A longer hospitalization may help reach symptom remission and prevent relapse, but it also further detaches the youth from their communities, peers and the usual educational and social activities, while being exposed to the potential iatrogenic effects of inpatient settings. A longer length of stay was also significantly associated with increased odds of seclusion or restraint (OR 1.01 per day) in a case-control study of inpatient admissions to a child and adolescent psychiatric unit. The risks were higher for youth with PTSD, borderline personality disorder, ADHD/conduct disorder, manic or bipolar episodes, and those in out-of-home care—highlighting the heightened risk for these vulnerable groups [36]. Long hospitalizations obviously contribute to the shortage of hospital beds that is seen in some areas. A too short hospitalization without appropriate aftercare, when necessary, in an intensive outpatient setting may increase the risk of re-hospitalization or major injuries or death after discharge [37].

In conclusion, the wide heterogeneity in youth psychiatric hospital beds in Europe is likely a reflection of major differences in the overall organization of mental health care for young people across the different countries. The roots of this diversity may be in differences in the historical development of child and adolescent psychiatry in each country, with its own medical tradition, education and training programs. In some cases, substantial within-country heterogeneity is also seen, pointing to the relevance of the regionally administered care systems. The striking differences in hospital beds do not seem to be linked to easily detectable differences in indexes of population mental health or behavioral functioning. This may be due to the lack of appropriate methods of ascertainment. The heterogeneity across countries that share much in common could be seen as research opportunity to learn through systematic observational studies, or quasi-experimental designs in cases of important policy changes in some countries but not in others, to learn about the impact of acute psychiatric care on both proximal outcomes, such as re-hospitalization, and more distal one, such as functional recovery and social inclusion. In the meantime, it would be helpful to have European recommendations for minimum requirements for acute child and adolescent psychiatric care, particularly for the treatment of suicidal crises, schizophrenia and other severe mental illness.

## Data Availability

No datasets were generated or analyzed during the current study.
